# Epidemiological distribution of genotypes of *Giardia duodenalis* in humans in Spain

**DOI:** 10.1186/s13071-019-3692-4

**Published:** 2019-09-06

**Authors:** Yuanfei Wang, Olga Gonzalez-Moreno, Dawn M. Roellig, Laura Oliver, Jordi Huguet, Yaqiong Guo, Yaoyu Feng, Lihua Xiao

**Affiliations:** 1 State Key Laboratory of Bioreactor Engineering, School of Resources and Environmental Engineering, East China University of Science and Technology, Shanghai, 200237 China; 2 Laboratory of Microbiology and Parasitology, SYNLAB, 08950 Barcelona, Spain; 3 Laboratory of Parasitology, Department of Biology, Healthcare and Environment, Faculty of Pharmacy, University of Barcelona, 08028 Barcelona, Spain; 4 Division of Foodborne, Waterborne, and Environmental Diseases, Centers for Disease Control and Prevention, Atlanta, Georgia 30333 USA; 5 Key Laboratory of Zoonosis of Ministry of Agriculture, College of Veterinary Medicine, South China Agricultural University, Guangzhou, 510642 China

**Keywords:** *Giardia duodenalis*, Multilocus genotyping, Assemblage, Spain

## Abstract

**Background:**

Although the distribution of *Giardia duodenalis* genotypes in humans has been increasingly reported in recent years, data on possible differences in pathogen transmission between age groups and virulence between genotypes are scarce. The purpose of this study is to investigate the genetic diversity of *G. duodenalis* in humans in Spain and compare the distribution of *G. duodenalis* assemblages A and B between children and adults and clinical presentations between the two genotypes.

**Methods:**

In the present study, 125 microscopy-positive fecal samples were collected from humans in Spain over a 7-year period. PCR and sequence analyses of the triosephosphate isomerase, β-giardin and glutamate dehydrogenase genes were used to identify the multilocus genotypes of *G. duodenalis*.

**Results:**

Sequence analysis of three genetic loci identified both *G. duodenalis* assemblages A (29) and B (66), with co-infections of the two in two patients. Among the sequences obtained in this study, four multilocus genotypes (MLGs) of the sub-assemblage AII were observed within assemblage A. In contrast, 19 MLGs were detected within assemblage B due to the high sequence diversity at each locus. One MLG, however, was found in 51.9% (27/52) of assemblage B samples. Children were more commonly infected by assemblage B (44/53 or 83%) than adults (22/42 or 52.4%; *χ*^2^ = 10.371, *df* = 1, *P* = 0.001). Asymptomatic infection was more common in patients with assemblage A (4/29 or 13.8%) than in those with assemblage B (1/66 or 1.5%; *χ*^2^ = 6.091, *df* = 1, *P* = 0.029), and the frequency of abdominal pain occurrence was higher in assemblage B patients (65/66 or 98.5%) than assemblage A patients (25/29 or 86.2%; *χ*^2^ = 6.091, *df* = 1, *P* = 0.029).

**Conclusions:**

These results illustrate the existence of differences in genotype distribution between children and adults and clinical presentations between *G. duodenalis* genotypes. They are useful in understanding the transmission of *G. duodenalis* in humans in Spain.

## Background

*Giardia duodenalis* is a common gastrointestinal pathogen in a wide range of vertebrates, including humans and domestic animals [1]. As one of the most common enteric pathogens, *G. duodenalis* is mainly transmitted through contact with infected persons or ingestion of contaminated food or water [2]. It colonizes the upper small intestine, causing asymptomatic, acute or chronic infections [3]. Worldwide, it is estimated that 280 million people are infected by *G. duodenalis* every year [4]. Human infection rates are generally high in developing countries, with pre-school children having the highest infection rates [1, 5]. Infection rates are also higher in children in industrialized nations, although older people can also have symptomatic *G. duodenalis* infections [6]. Symptoms of giardiasis include diarrhea, nausea, vomiting, abdominal pain, bloating, epigastric pain and weight loss [2, 7], which can develop 6–15 days after infection [8]. In developing countries, giardiasis has been further associated with growth retardation and poor cognitive performance in children [9].

*Giardia duodenalis* is a multispecies complex with at least eight recognized assemblages or genotypes (A-H) based on the genetic characterization of pathogens in clinical specimens [10, 11]. Among them, assemblages A and B are major human pathogens [1]. Assemblage A is further detected in livestock and companion animals, which are more often infected with their own host-adapted genotypes (assemblages C-F). In contrast, assemblage B is commonly reported in only a small number of animal species as the dominant *G. duodenalis* genotype [1]. Evidence for the occurrence of zoonotic transmission has come from the finding of the same subtypes or multilocus genotypes (MLGs) in humans and animals in the same area. For example, some human isolates of sub-assemblage AI clustered together with animal isolates in a recent study in Sweden [12]. Thus, both assemblages A and B are considered zoonotic pathogens, although the significance of zoonotic infection in giardiasis epidemiology remains unclear [1]. In addition, a small number of infections with assemblage E have been reported in humans in Australia, Brazil and Egypt [13].

Molecular analysis of more than 2800 *G. duodenalis*-positive samples from humans indicates that assemblage B (accounting for ~ 58% giardiasis cases) has a higher prevalence than assemblage A (accounting for ~ 37% giardiasis cases) worldwide [14]. The distribution of the two assemblages, however, differs among areas. For example, only assemblage A was detected in some studies in Uganda, USA, Canada and Korea [15], only assemblage B was detected in a study in India [16], while similar occurrence of assemblages A and B was identified studies of giardiasis in Slovenia, Netherlands and Albania [15, 17, 18]. Some previous studies carried out in Spain demonstrated a different distribution of *G. duodenalis* assemblages among some provinces. For instance, assemblages A and B were detected in 27.4 and 72.6%, respectively, of *G. duodenalis-*positive patients in Madrid [19], while an equal distribution of assemblages A and B was identified in La Rioja [20].

Although *G. duodenalis* is a common human pathogen worldwide, few data are available on differences in pathogen transmission between children and adults and clinical presentations between genotypes. One study in Australia indicated that adults were more commonly infected with *G. duodenalis* assemblage A compared with children [21]. Differences in virulence between assemblages A and B have been reported, with assemblage B being more virulent in most recent reports and more common in outbreaks [13]. In Spain, the few recent molecular epidemiological surveys indicate that assemblage B is the dominant *G. duodenalis* in humans [19, 20, 22]. The infection rates varied from 3.1% to 17.8% among these Spanish studies [20, 23, 24]. However, only one or two of the three commonly used genotyping loci, namely β-giardin (*bg*), glutamate dehydrogenase (*gdh*) and triosephosphate isomerase (*tpi*), were used in these studies. In addition, all samples in each survey were collected from the same city.

To fill some of the knowledge gaps described above, in the present study, multilocus genotyping targeting the three classical genetic loci (*bg*, *gdh* and *tpi*) was used to characterize *G. duodenalis* in clinical samples collected from humans in multiple locations in Spain between 2012 and 2018. The distribution of *G. duodenalis* genotypes was compared between children and adults and clinical presentations compared between assemblages A and B to assess the possibility of differences in the transmission of *G. duodenalis* between the two populations and virulence between the two assemblages.

## Methods

### Sample collection

A total of 125 fecal samples positive for *G. duodenalis* by microscopy were used in this study. They were from out-patients in hospitals in 10 Spanish provinces during 2012 (*n* = 9), 2015 (*n* = 27), 2016 (*n* = 33), 2017 (*n* = 25) and 2018 (*n* = 31) and submitted to a commercial laboratory, Synlab Diagnosticos Globales (Barcelona, Spain), for the detection of enteric pathogens, including common viruses, bacteria and parasites; samples positive for these bacteria and viruses were not further tested for enteric parasites. Each patient was supplied with one or more sterile plastic containers (1–3) with 5 ml of merthiolate-iodine-formaldehyde (MIF) solution to preserve the fecal samples; these were kept at room temperature until they were examined by microscopy. Among them, 118 were from patients suffering from diarrhea or other intestinal symptoms, while 7 were from asymptomatic persons who requested testing specifically for *G. duodenalis* because of unknown reasons. The patients were 1–75 years of age, with a median age of 10 years, and comprised 67 males and 58 females.

### Microscopy analysis of *G. duodenalis*

The fecal samples were analyzed for *G. duodenalis* by direct microscopic examination of wet mount of fecal materials fixed with MIF solution as previously described [25]. Briefly, wet mounts were made using 20 μl of the upper suspension of the MIF-fixed sample. The entire area of the wet mounts was examined under a BX50 light microscope (Olympus, Tokyo, Japan) using 20× and 40× objectives. The samples positive for *G. duodenalis* were washed by centrifugation and stored in 70% ethanol at 4 °C before being shipped to the laboratory at the Centers for Disease Control and Prevention for molecular analysis.

### DNA extraction and PCR amplification

Approximately 500 μl of suspension of the ethanol-preserved fecal sample was washed twice with distilled water by centrifugation at 2000× *g* for 10 min to remove ethanol. Genomic DNA was extracted from the washed fecal material using a FastDNA SPIN Kit for Soil (MP Biomedicals, Santa Ana, CA, USA) as described [26] and stored at − 80 °C until being analyzed by PCR within six months.

To identify MLGs of *G. duodenalis*, a 532-bp fragment of the *tpi* gene, a 511-bp fragment of the *bg* gene and a 530-bp fragment of the *gdh* gene were amplified using nested PCR [1]. To reduce the effect of residual PCR inhibitors in the extracted DNA, 400 ng/μl of non-acetylated bovine serum albumin (Sigma Aldrich, St. Louis, MO, USA) was used in primary PCR. Each DNA sample was analyzed in duplicate, using both positive (assemblage E) and negative (reagent-grade water) controls in each analysis. The secondary PCR products were visualized by 1.5% agarose gel electrophoresis.

### Sequence analysis

All positive secondary PCR products from the three genetic loci generated in the study were sequenced in both directions using the secondary primers and the BigDye Terminator v.3.1 Cycle Sequencing Kit (Applied Biosystems, Foster City, CA, USA) on an ABI 3730 Genetic Analyzer (Applied Biosystems). The nucleotide sequences of each gene were assembled using ChromasPro v.1.32 (http://technelysium.com.au/ChromasPro.html) and aligned with reference sequences (U57897, AY072723, AY072724, AY178737 and EF507651 for assemblage A, and KX468986, L02116, EU272153, FJ560565, HM140723, AB781124, KX960128, EU637581, KX469029, KJ888980, AY072728, HM165218, AY072727, EF507671, EU834843, KY696790 and KM977636 for assemblage B) downloaded from the GenBank database using the software ClustalX (http://www.clustal.org/) to identify the genotypes and subtypes of *G. duodenalis*. The evolution of assemblage B MLGs was assessed by using eBURST v.3.0 (http://eBURST.mlst.net/). Samples with apparent presence of double peaks at any of the loci during DNA sequencing were excluded in the MLG analysis of sequence data.

### Data analysis

The Chi-square test was used to compare differences in infection rates of assemblages among children and adult and clinical symptoms. Differences were considered significant at the level of *P* < 0.05. The probability for the occurrence of asymptomatic infection or abdominal pain between assemblages A and B was measured by the odds ratio (OR) together with its 95% confidence intervals (95% CI). Statistical analysis was performed using SPSS Statistics v.21.0 for Windows (IBM Corp., Armonk, NY, USA).

## Results

### Detection and identification of *G. duodenalis* genotypes

**Fig. 1:**
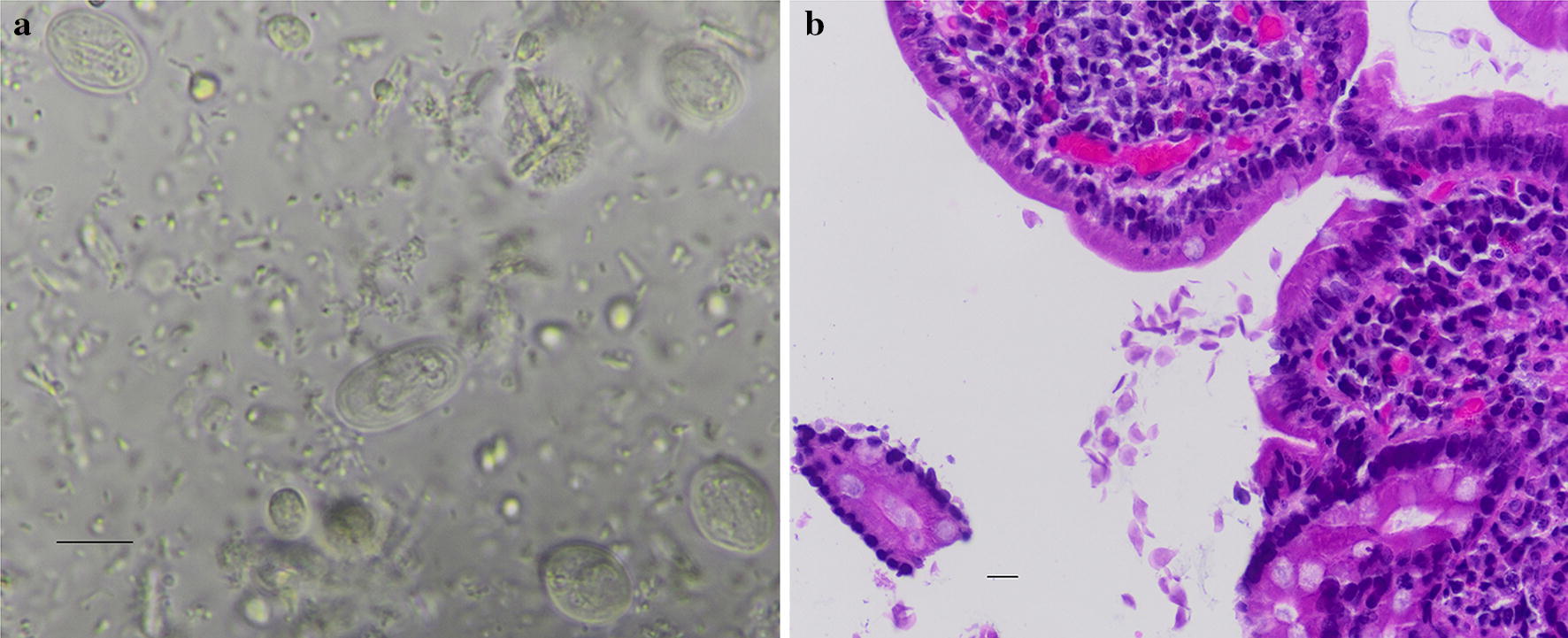
Cysts (**a**) and trophozoites (**b**) of *Giardia duodenalis* in a fecal sample and a duodenum biopsy of patients in Spain, respectively. The cysts were detected in microscopic analysis of wet mount of fecal material fixed with the merthiolate-iodine-formaldehyde solution under an Olympus BX43 using a 100× objective, while the trophozoites were detected by microscopic analysis of hematoxylin-eosin stained tissue section under an Olympus BX43 using a 60× objective. *Scale-bars*: 10 µm

Altogether, 125 *G. duodenalis*-positive fecal samples were included in this study. They were mostly diagnosed by microscopy detection of cysts in fecal samples (Fig. 1a). One fecal sample, however, was collected from a patient who was initially diagnosed as positive for *G. duodenalis* based on the detection of trophozoites in duodenum biopsies (Fig. 1b). Twelve samples collected during the period that had co-infections with other enteric protozoa, including *Blastocystis hominis* (*n* = 11), *Endolimax nana* (*n* = 2), *Entamoeba coli* (*n* = 2), *Cryptosporidium* sp. (*n* = 1) and *Iodamoeba butschlii* (*n* = 1). They were not included in the analysis of the associations between symptoms and assemblages of *G. duodenalis*.

Among the 125 fecal samples, 97 were positive in PCR analyses of the three genetic loci. The number of PCR-positive samples were 89, 84 and 87 at the *tpi*, *bg* and *gdh* loci, respectively. The amplification rates were 8/9 (88.9%), 23/27 (85.2%), 25/33 (75.8%), 20/25 (80%) and 21/31 (67.7%) for samples collected during 2012, 2015, 2016, 2017 and 2018, respectively. Among the 97 genotyped samples, 78 were successfully sequenced at all three genetic loci; the remaining 19 samples were successfully genotyped at only one (*n* = 12) or two (*n* = 7) genetic loci. Two genotypes of *G. duodenalis* were detected among the 97 genotyped samples, namely assemblages A (29, 30%) and B (66, 68%), with two samples having both (Table 1). Between the two samples with mixed genotypes, one was identified as assemblage B at the *tpi* locus but assemblage A at *bg* and *gdh* loci, while the other was identified as assemblage B at the *gdh* locus but assemblage A at *tpi* and *bg* loci.

**Table 1:** Occurrence of *Giardia duodenalis* genotypes (assemblages A and B) by age, gender, geographical source and symptom of the 125 microscopy-positive patients enrolled in the study in Spain during 2012–2018

Group	Case count	No. PCR-positive	Assemblage			Clinical symptom							
			A	B	A + B	Abdominal pain	Bloating	Diarrhea	Nausea	Dehydration	Weight loss	Epigastric pain	Normal
Age (years)													
0–5	46	38	6	31	1	37 (44)	19 (24)	7 (8)	3 (3)	2 (2)	2 (2)	0	1 (2)
6–12	22	16	3	13	0	16 (21)	10 (13)	2 (2)	1 (1)	0	1 (1)	0	0 (1)
23–75	57	43	20	22	1	39 (53)	11 (16)	10 (14)	0	0	1 (1)	5 (6)	4 (4)
Gender													
Male	67	55	15	39	1	52 (62)	23 (29)	9 (13)	3 (3)	1 (1)	1 (1)	3 (3)	3 (5)
Female	58	42	14	27	1	40 (56)	17 (24)	10 (11)	1 (1)	1 (1)	3 (3)	2 (3)	2 (2)
Geographical source													
Barcelona	87	72	24	46	2	69 (83)	28 (33)	12 (13)	3 (3)	1 (1)	2 (2)	3 (3)	3 (4)
Málaga	13	8	3	5	0	7 (12)	5 (9)	1 (2)	0	0	1 (1)	1 (1)	1 (1)
Mallorca	6	5	1	4	0	5 (6)	2 (2)	0	0	0	0	1 (2)	0
Madrid	6	4	0	4	0	4 (6)	3 (5)	3 (5)	0	0	0	0	0
Tarragona	5	4	1	3	0	4 (4)	1 (1)	1 (1)	0	0	1 (1)	0	0 (1)
Sevilla	3	2	0	2	0	2 (3)	1 (2)	1 (1)	0	0	0	0	0
Girona	2	1	0	1	0	0 (1)	0	0	0	0	0	0	1 (1)
Cadiz	1	0	0	0	0	0 (1)	0	0	0	0	0	0	0
Valladolid	1	1	0	1	0	1 (1)	0	1 (1)	1 (1)	1 (1)	0	0	0
Badajoz	1	0	0	0	0	0 (1)	0 (1)	0 (1)	0	0	0	0	0
Subtotal	125	97	29	66	2	92 (118)	40 (53)	19 (24)	4 (4)	2 (2)	4 (4)	5 (6)	5 (7)

*Note*: Numbers in parentheses are the total numbers of samples examined, while those without parentheses are numbers of PCR-positive samples

### Distribution of *G. duodenalis* genotypes by age, gender, location and clinical symptom

Among the 125 microscopy-positive samples, 46 (36.8%), 22 (17.6%) and 57 (45.6%) were from the age groups of 0–5, 6–12 and 23–75 years, respectively (Table 1). The numbers of PCR-positive samples from the three age groups were 38, 16 and 43, respectively. There was a significant difference in the distribution of *G. duodenalis* assemblages between children and adults (*χ*^2^ = 10.371, *df* = 1, *P* = 0.001). Children under 12 years-old were more commonly infected by assemblage B (44/53 or 83.0%) than assemblage A (9/53 or 17.0%). In contrast, adults had similar distribution of assemblages A (20/42 or 47.6%) and B (22/42 or 52.4%). Among the 97 PCR-positive samples, 55 and 42 were from male and female patients, respectively. There was no significant difference in the distribution of *G. duodenalis* assemblages A and B between male (15/54 or 27.8% and 39/54 or 72.2%, respectively) and female (14/41 or 34.1% and 27/41 or 65.9%, respectively) patients (*χ*^2^ = 0.446, *df* = 1, *P* = 0.328).

Eighty-seven (69.6%) of the 125 microscopy-positive samples were collected from Barcelona. Among them, 72 samples were PCR-positive for *G. duodenalis*, including 24 and 46 samples identified as assemblages A and B, respectively (two samples had both). The remaining 38 samples (30.4%) were collected from 9 other provinces, with 25 of them being PCR-positive. Among the latter, 5 and 20 samples were identified as assemblages A and B, respectively.

Abdominal pain was the most common clinical symptom of giardiasis: 118 (94.4%) of the 125 patients had the symptom (Table 1). The other two main clinical manifestations included bloating and diarrhea: 53 and 24 of the study patients, respectively, had these symptoms. Other clinical manifestations such as nausea, dehydration, weight loss and epigastric pain were observed in 4, 2, 4 and 6 patients, respectively. In addition, 7 patients were asymptomatic at the time of sampling.

The distribution of *G. duodenalis* genotypes by clinical symptom is shown in Table 2. Patients infected with assemblages A and B had a different frequency of asymptomatic infection (*χ*^2^ = 6.091, *df* = 1, *P* = 0.029). Therefore, assemblage A-infected patients (4/29 or 13.8%) were more likely to have asymptomatic infection than assemblage B-infected patients (1/66 or 1.5%) (OR: 10.4, 95% CI: 1.108–97.625). In addition, the frequency (% occurrence) of abdominal pain was significantly different in patients infected by assemblages A and B (*χ*^2^ = 6.091, *df* = 1, *P* = 0.029). Among the 29 assemblage A-infected patients, 25 (86.2%) had abdominal pain. In contrast, among the assemblage B-infected patients, 65 of 66 (98.5%) had this symptom. Other clinical symptoms such as bloating, diarrhea, nausea, dehydration, weight loss and epigastric pain were equally distributed between patients infected with the two assemblages (Table 2).

**Table 2:** Distribution of clinical symptoms in 97 PCR-positive patients enrolled in the study by assemblage (A or B) of *Giardia duodenalis*

Group	Case count	Clinical symptom							
		Abdominal pain	Bloating	Diarrhea	Nausea	Dehydration	Weight loss	Epigastric pain	Normal
Assemblage A	29	25	9	5	1	0	1	3	4
Assemblage B	66	65	30	13	3	1	3	2	1
Assemblage A + B	2	2	1	1	0	1	0	0	0
Subtotal	97	92	40	19	4	2	4	5	5

### Distribution of assemblage A subtypes

Within assemblage A, only the A2 subtype was detected in 27 (100%) assemblage A-positive samples at the *tpi* locus, with the sequence being identical to U57897 in GenBank. In contrast, three subtypes were observed among the 28 assemblage A-positive samples at the *bg* locus, with sequences being identical to AY072723 (A2) in 10 (35.7%) samples, AY072724 (A3) in 17 (60.7%) samples and AB469365 (A5) in one (3.6%) sample. Two subtypes were observed at the *gdh* locus among the 27 assemblage A-positive samples, with the sequences being identical to AY178737 (A2) in 9 (33.3%) samples and EF507651 (A4) in 18 (66.7%) samples (Table 3).

**Table 3:** Distribution of multilocus genotypes of *Giardia duodenalis* in 76 patient samples with complete data at all three genetic loci in the study conducted in Spain during 2012–2018

Sample ID	Genotype (GenBank ID)			MLG type (*n*)
	*tpi*	*bg*	*gdh*	
38808, 42849, 42853, 42855, 44333, 44945, 45105, 45598, 45599, 45606	A2 (U57897)	A3 (AY072724)	A2 (AY178737)	AII-9 (10)
42838, 42844, 44335, 44347, 44944, 45610, 45751	A2 (U57897)	A2 (AY072723)	A2 (AY178737)	AII-1 (7)
44943, 44948, 44949, 45608, 45611, 45745	A2 (U57897)	A3 (AY072724)	A4 (EF507651)	AII-4 (6)
45612	A2 (U57897)	A2 (AY072723)	A4 (EF507651)	AII-8 (1)
38810, 38811, 42835, 42837, 42845, 42848, 42857, 42858, 42863, 44329, 44330, 44332, 44334, 44338, 44343, 44357, 44358, 44359, 44361, 44947, 45099, 45100, 45102, 45103, 45602, 45609, 45756	B (KX468986)	B2 (KX960128)	BIV (EF507671)	MLG B1 (27)
42860, 44939, 44940, 45107, 45600	B (KX468986)	B1 (EU637581)	B3 (EU834843)	MLG B2 (5)
38807, 44354, 44355	B (KX468986)	B2 (KX960128)	B3 (EU834843)	MLG B3 (3)
38814, 45607	B (L02116)	B (MG754396)	B3 (EU834843)	MLG B4 (2)
45613	B (KX468986)	B2 (KX960128)	KY696790-B	MLG B5 (1)
42846	B (KX468986)	B4 (KX469029)	BIV (EF507671)	MLG B6 (1)
44356	B (KX468986)	B (KJ888980)	BIV (EF507671)	MLG B7 (1)
45106	B (KX468986)	B (AY072728)	B3 (EU834843)	MLG B8 (1)
45101	B (KX468986)	B (MG754396)	B (KY696790)	MLG B9 (1)
45104	B (KX468986)	B (MG754396)	B3 (EU834843)	MLG B10 (1)
38815	B (L02116)	B1 (EU637581)	BIV (EF507671)	MLG B11 (1)
45604	B (L02116)	B1 (EU637581)	B3 (EU834843)	MLG B12 (1)
44352	B (L02116)	B2 (KX960128)	B3 (EU834843)	MLG B13 (1)
42834	B (L02116)	B2 (KX960128)	BIV (EF507671)	MLG B14 (1)
38813	B (L02116)	B (MG754395)	B (MG754398)	MLG B15 (1)
45755	B (L02116)	B (AY072727)	BIV (EF507671)	MLG B16 (1)
42841	B (EU272153)	B (AY072728)	B2 (KM977636)	MLG B17 (1)
42862	B (FJ560565)	B6 (HM165218)	BIV (EF507671)	MLG B18 (1)
44351	B (HM140723)	B (MG754396)	B3 (EU834843)	MLG B19 (1)

### Distribution of assemblage B subtypes

More subtypes were detected among assemblage B samples at each of the three genetic loci (Table 3 and Additional file 1: Table S1). Compared with the reference sequence KX468986, 1–5 single nucleotide substitutions (SNPs) were detected at eight positions at the *tpi* locus among assemblage B samples in this study. Altogether, seven subtypes of assemblage B were observed at the *tpi* locus, 6 of which were identical to KX468986, L02116, EU272153, FJ560565, HM140723 and AB781124 in the GenBank. The remaining one had 99% sequence identity to KX469015, was named as TB1, and deposited in GenBank under the accession number MG754394. Among them, the KX468986 sequence type was the dominant subtype, being detected in 47 of 66 assemblage B-positive samples. The second most common sequence type was L02116, which was detected in 9 samples. The remaining 5 sequence types were only detected in one or two samples during the five years of study.

Similarly, compared with the reference KX960128, 10 subtypes were observed at the *bg* locus among the 66 assemblage B samples, including three novel subtypes (Table 3 and Additional file 1: Table S1). The latter were named as BB1, BB2 and BB3, with sequences being deposited in GenBank under accession numbers MG754395–MG754397. Among the assemblage B samples, KX960128-B2 was the dominant subtype at the *bg* locus, being detected in 36 samples. This was followed by EU637581-B1, which was detected in 7 samples. The remaining subtypes were detected in 1–5 samples.

At the *gdh* locus, seven subtypes were observed among 66 assemblage B samples (Table 3 and Additional file 1: Table S1). Compared with the reference EF507671, 1–11 SNPs were detected at 19 positions, with three novel subtypes being identified. The latter were named as GB1, GB2 and GB3, with sequences being deposited in GenBank under the accession numbers MG754398, MG754399 and MG767308. Among the assemblage B samples, EF507671-BIV was the dominant subtype, being detected in 33 samples. This was followed by EU834843-B3, which was detected in 21 samples. The remaining subtypes were each observed in only one or two samples.

### Distribution of *G. duodenalis* MLGs

Altogether, four MLGs were presented among the 24 assemblage A samples that were successfully subtyped at all three genetic loci (Table 3). Among them, AII-9 was the dominant MLG, being detected in 10 samples. The remaining MLGs, including AII-1, AII-4 and AII-8, were detected in 7, 6 and 1 samples, respectively.

In contrast, 19 MLGs were detected among the 52 assemblage B samples that were successfully subtyped at all three genetic loci (Table 3). Among them, MLG-B1 was the dominant type, being detected in 27 (51.9%) samples. MLG-B2, MLG-B3 and MLG-B4 were less frequent, being detected in 5, 3 and 2 samples, respectively. The remaining 15 MLGs (MLG-B5 to MLG-B19) were each detected in only one samples.

**Fig. 2:**
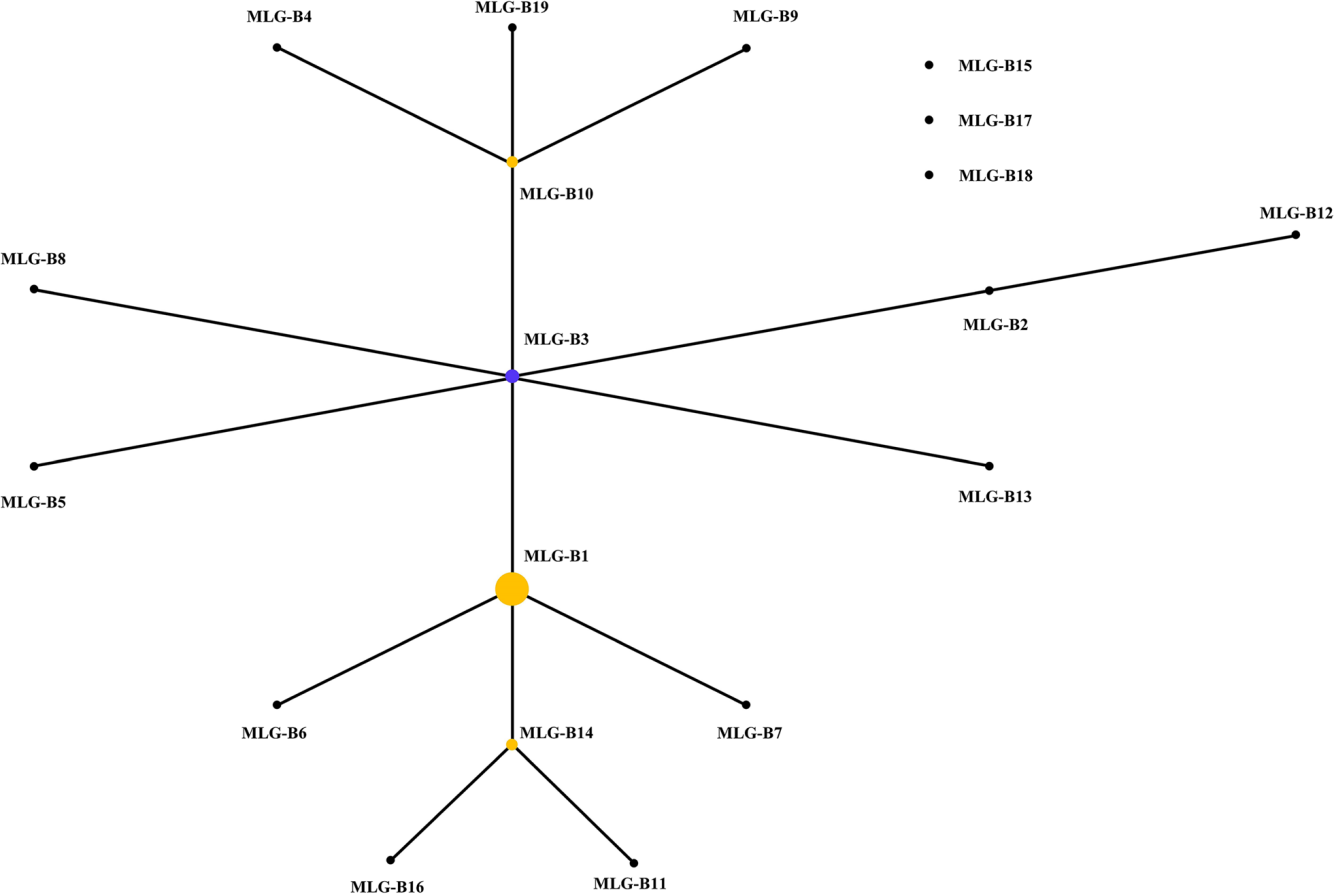
Genetic relationship of multilocus genotypes (MLGs) of *Giardia duodenalis* assemblage B based on eBURST analysis of sequences of the triosephosphate isomerase, β-giardin, and glutamate dehydrogenase genes. Each MLG is represented by a dot. The size of each dot is proportional to the number of samples with the MLG. The primary founder of the group is colored blue, while the subgroup founder is colored yellow. Single-locus variants are connected by lines. MLG-B15, MLG-B17 and MLG-B18 form singletons outside the main cluster

In the eBURST analysis of the MLG data from assemblage B, one cluster and three singletons of MLGs were observed. In the cluster, MLG-B3 was the primary founder and other common MLGs, such as MLG-B1, MLG-B10 and MLG-B14, were subgroup founders. The most commonly detected MLG, MLG-B1, appeared to be a single locus variant of the primary founder MLG-B3 (Fig. 2).

## Discussion

In the present study, we assessed the genetic diversity of *G. duodenalis* in humans in Spain during 2012–2018. Among the 125 microscopy-positive samples collected from out-patients in ten provinces, 97 (77.6%) generated the expected products in PCR. The genotyping results obtained suggest the occurrence of both assemblages A and B in Spain, with assemblage B (66/95 or 69.5%) being twice as common as assemblage A (29/95 or 30.5%). No assemblage E infection was detected in this study. This is consistent with observations in three previous studies in Spain, which had also shown a more frequent occurrence of assemblage B than assemblage A in humans in Zaragoza, Madrid and La Rioja provinces [19, 20, 22]. The failure in PCR amplification of DNA from some microscopy-positive samples could be due to the initial short storage of the fecal material in MIF, which contains PCR-unfriendly preservative formalin; PCR is known to be more sensitive than conventional microscopy in detecting *G. duodenalis* [27, 28].

The distribution of *G. duodenalis* genotypes appears to be different between children and adults in Spain. In this study, 83% (44/53) of *G. duodenalis*-positive children were infected with assemblage B, compared with 52.4% (22/42) in adults (*χ*^2^ = 10.371, *df* = 1, *P* = 0.001). Similar results were obtained in one previous study in Egypt, where children had a higher frequency of assemblage B (24/34 or 70.6%) than assemblage A (10/34 or 29.4%), while adults had a similar frequency of both assemblages (12/26 or 46.2% and 14/26 or 53.8%, respectively) [29]. In contrast, the opposite was observed in a study conducted in the UK in which assemblages A and B were equally distributed in children of 0–9 years, assemblage B was more common in adults of 30–49 years, while assemblage A was more common in adults over 50 years [30]. These results indicate that the dominant genotypes of *G. duodenalis* could change in humans over age, perhaps as a reflection of the development of acquired immunity and/or differences in exposures.

The clinical symptoms caused by assemblages A and B appear to be different in the Spanish population. In the present study, abdominal pain was the most common clinical symptom, but patients infected by *G. duodenalis* assemblage B were more likely to develop it than patients infected by assemblage A (OR: 10.4, 95% CI: 1.108–97.625). This is different from data from the UK, in which the frequency of abdominal pain occurrence was similar between patients infected with the two assemblages [31]. In addition, assemblage A-infected patients were more likely to have asymptomatic infections in our study (OR: 10.4, 95% CI: 1.108–97.625), which is similar to observations in previous studies in Ethiopia, Netherlands and Argentina [32–35]. However, in another study of giardiasis in children under five years in Spain, symptoms were more common in patients infected by assemblage A than by assemblage B [22]. Similarly, assemblage B-infected patients were more likely to have vomiting than assemblage A-infected patients in a study in England [30], while there was no significant difference in the occurrence of vomiting between infections with assemblages A and B in the present study. Therefore, multiple factors could affect the clinical presentations of *G. duodenalis* genotypes.

Anthroponotic transmission appears to play an important role in giardiasis epidemiology in Spain. In the present study, almost all assemblage A-positive samples were identified as having sub-assemblage AII, which is known to preferentially infect humans [1]. This is similar to previous observations in South America [36, 37]. In addition, assemblage B was the dominant genotype in this study, which is consistent with data from South America and Africa [5, 35, 38, 39]. Assemblage B detected in these studies has only occasionally been detected in farm animals or companion animals. Therefore, both observations above suggest that *G. duodenalis* in the Spanish population could be mostly anthroponotic in origin.

In the present study, a higher genetic heterogeneity was observed in assemblage B than in assemblage A. Among the latter, most of the cases except one belonged to three MLGs within the sub-assemblage AII. In contrast, 19 MLGs were detected among assemblage B isolates, which is consistent with previous observations [30, 40, 41]. The identification of MLGs within assemblage B is complicated by the possible presence of sequence heterozygosity at some of the genetic loci, which may lead to the over-estimation of MLGs [19, 40]. Therefore, meiotic recombination, the presence of sequence heterozygosity and concurrence of mixed subtypes could all contribute to the high MLG numbers within assemblage B [42, 43]. Nevertheless, in agreement with the observation with assemblage A in this study, over half (27/52 or 51.9%) of the assemblage B infections in the Spanish population were caused by one MLG, indicating the possible circulation of one subtype in the community, and the occurrence of clonal expansion of limited number of subtypes, a finding in agreement with the result of the eBURST analysis (Fig. 2).

## Conclusions

Results of the MLG analysis demonstrate a common occurrence of both assemblages A and B of *G. duodenalis* in the Spanish population, with a different distribution of the two genotypes between children and adults and some genotype-associated differences in clinical presentations or virulence. Despite the observation of high genetic diversity in *G. duodenalis* at the subtype level, several MLGs appear to have high occurrence in the human populations examined. The genetic identity of *G. duodenalis* at both the genotype and subtype levels suggests that anthroponotic transmission is important giardiasis epidemiology in the study area. Further studies using a better epidemiological design and more advanced molecular biologic tools are, however, needed to confirm these hypotheses.

## Supplementary information

**Additional file 1: Table S1.** Distribution of *Giardia duodenalis* subtypes in 21 human samples with partial or discrepant data at three genetic loci in Spain.

## Data Availability

The data supporting the conclusions of this article are included within the article and its additional file. Representative nucleotide sequences generated in this study were submitted to the GenBank database under the accession numbers MG754394–MG754399 and MG767308.

## Abbreviations

PCR: polymerase chain reaction
MLGs: multilocus genotypes
*tpi*: triosephosphate isomerase
*bg*: β-giardin
*gdh*: glutamate dehydrogenase
SNPs: single nucleotide substitutions
