# Supplementary material for: Epidemiological distribution of genotypes of Giardia duodenalis in humans in Spain
Source: Parasit Vectors. 2019 Sep 6;12:432. doi: 10.1186/s13071-019-3692-4 (PMC6728964; doi:10.1186/s13071-019-3692-4)
Supplement: Supplementary file 1 — Additional file 1: Table S1. Distribution of Giardia duodenalis subtypes in 21 human samples with partial or discrepant data at three genetic loci in Spain. [file 13071_2019_3692_MOESM1_ESM.docx]

**Additional file**

**Additional file 1: Table S1** Distribution of *Giardia duodenalis* subtypes in 21 human samples with partial or discrepant data at three genetic loci in Spain

| **Specimen ID** | **Genotype (GenBank Accession No.)** | | |
| --- | --- | --- | --- |
|  | ***tpi*** | ***bg*** | ***gdh*** |
| 42864 | B (L02116) | A2 (AY072723) | A4 (EF507651) |
| 45601 | A2 (U57897) | A2 (AY072723) | B3 (EU834843) |
| 44936 | A2 (U57897) | A3 (AY072724) | - |
| 44360 | B (KX468986) | B2 (KX960128) | - |
| 42865 | B (KX468986) | B (MG754397) | - |
| 45605 | B (KX468986) | - | B3 (EU834843) |
| 44937 | B (HM140723) | - | B3 (EU834843) |
| 45098 | B (AB781124) | - | B (MG767308) |
| 44353 | - | B2 (KX960128) | B3 (EU834843) |
| 45757 | A2 (U57897) | - | - |
| 44342 | - | - | A2 (AY178737) |
| 42843 | - |  | A4 (EF507651) |
| 44350 | - | A5 (AB469365) | - |
| 42839, 44339, 44345 | B (KX468986) |  | - |
| 42840 | - | B2 (KX960128) | - |
| 38812 | B (MG754394) | - | - |
| 42859, 45753 | - | - | B3 (EU834843) |
| 44348 | - | - | B (MG754399) |
